# Unwilling or unable? Interpreting effort task performance in myalgic encephalomyelitis/chronic fatigue syndrome

**DOI:** 10.3389/fpsyg.2025.1593269

**Published:** 2025-06-13

**Authors:** Andrew Kirvin-Quamme, Karen D. Kirke, Oscar Junge, Jonathan C. W. Edwards, Kevin J. Holmes

**Affiliations:** ^1^Independent scholar, Boston, MA, United States; ^2^Independent scholar, Dublin, Ireland; ^3^Independent scholar, Amsterdam, Netherlands; ^4^Department of Medicine, University College London, London, United Kingdom; ^5^Department of Psychology, Reed College, Portland, OR, United States

**Keywords:** myalgic encephalomyelitis, chronic fatigue syndrome, ME/CFS, effort, effort preference, effort-based decision-making, calibration

## Introduction

In a recent, high-profile study of post-infectious myalgic encephalomyelitis/chronic fatigue syndrome (PI-ME/CFS), Walitt et al. (2024) assessed the performance of patients and healthy volunteers on the Effort-Expenditure for Rewards Task (EEfRT), among a host of other measures. The EEfRT is a widely used behavioral index of reward motivation and effort-based decision-making that requires repeatedly choosing between an easy task and a hard task, each involving rapid, repetitive button-pressing (Treadway et al., 2009). Walitt et al.'s study—the first to investigate effort-based decision-making in PI-ME/CFS—found that patients were less likely to choose the hard task than healthy volunteers. The authors interpreted this difference as evidence of altered “effort preference,” which they defined as “how much effort a person subjectively wants to exert” (p. 9). Walitt et al. concluded that “effort preference, not fatigue, is the defining motor behavior of this illness” (p. 10). Here we interrogate this conclusion. Were PI-ME/CFS patients less likely to choose the hard task because they *wanted* to exert less effort, consciously or otherwise? Or were they *less able* to complete the hard task, and thus chose it less often? We argue that the data support the latter interpretation.

## Accounting for ability in the EEfRT

For the EEfRT to yield interpretable results, participants' choices between the easy and hard tasks must be decoupled from their ability to complete these tasks. As the developers of the measure cautioned, “an important requirement for the EEfRT is that it measure individual differences in motivation for rewards, rather than individual differences in ability or fatigue” (Treadway et al., 2009, p. 4). Several techniques have been established to satisfy this requirement. In an initial validation study with a healthy student sample, Treadway et al. (2009) ruled out differences in both ability and within-task fatigue through two manipulation checks: confirmation of ceiling-level trial completion rates for all participants (96–100%) and the inclusion of trial number in statistical models. Subsequent studies have prospectively assessed and statistically controlled for motor skills, which have been shown to influence effort-based decision-making (Ohmann et al., 2020, 2022). In studies of schizophrenia, where patients have exhibited lower maximum button press rates than controls, the required number of button presses is often calibrated to individual ability levels (e.g., Cooper et al., 2019; Fervaha et al., 2013; Le et al., 2023; Reddy et al., 2015). This method helps fulfill the prerequisite, affirmed by the EEfRT developers, that “*all* subjects [are] *readily able* to complete both the hard and easy tasks *throughout* the experiment” (Treadway et al., 2009, p. 4; emphasis added).

## Walitt et al.'s interpretation: preference for avoiding effort

Despite the importance of ensuring that decision-making in the EEfRT cannot be explained by ability or fatigue, Walitt et al. (2024) only ruled out fatigue.^1^ In their study, 15 PI-ME/CFS patients and 16 healthy controls chose between the easy and hard tasks an average of 46 times during the 15-minute testing session. The easy task required 30 button presses in 7 seconds with the dominant index finger, while the hard task required 98 button presses in 21 seconds with the non-dominant little finger.^2^ Whereas the EEfRT is typically regarded as a measure of reward motivation, effort-based decision-making, or effort allocation (Cooper et al., 2019; Fervaha et al., 2013; Le et al., 2023; Ohmann et al., 2020, 2022; Reddy et al., 2015; Treadway et al., 2009), Walitt et al. characterized it as assessing the new construct of “effort preference, the decision to avoid the harder task when decision-making is unsupervised and reward values and probabilities of receiving a reward are standardized” (p. 2). Effort preference was operationalized as the Proportion of Hard Task Choices (PHTC). Compared to the PI-ME/CFS patients, the controls were 1.65 times more likely to choose the hard task (PHTC) and, upon choosing it, were 27.23 times more likely to successfully complete it. Walitt et al. interpreted the difference in PHTC as indicating that people with PI-ME/CFS prefer to avoid effort, ignoring the substantially larger—and more fundamental—difference in the ability of patients and controls to complete the hard task successfully.

## Our interpretation: limited ability to execute the task

**Figure 1:**
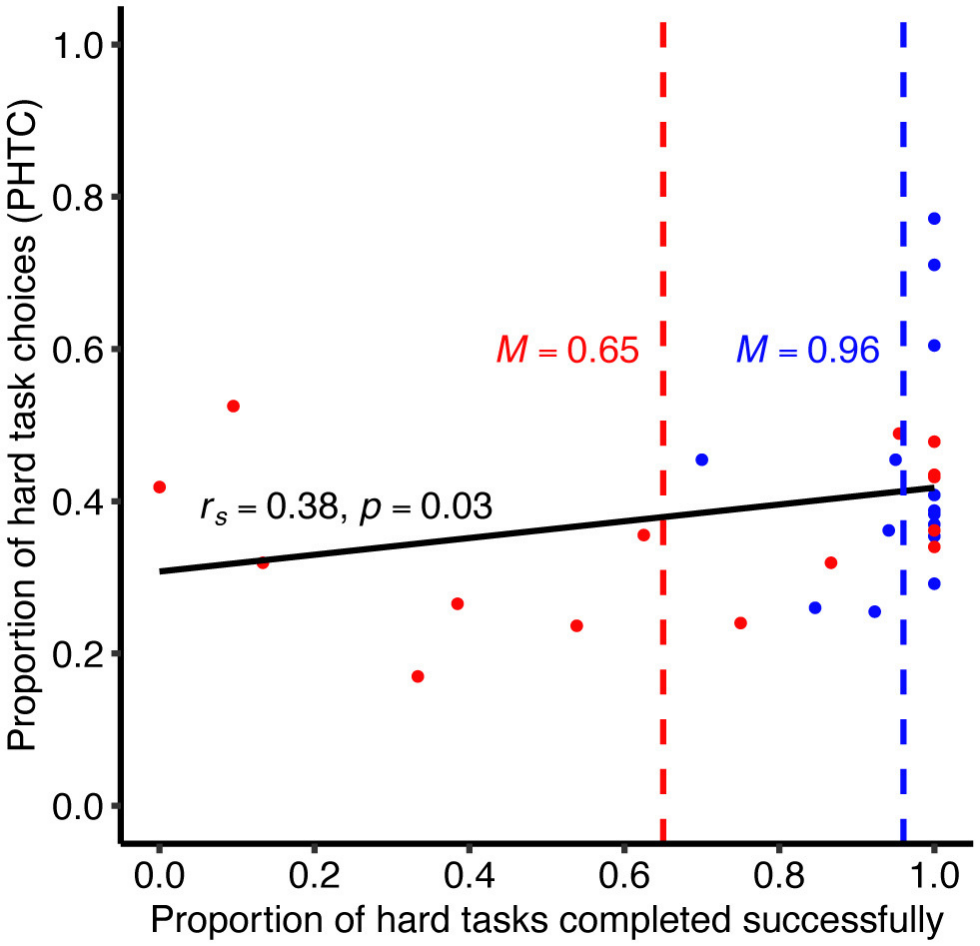
Positive correlation between the proportion of hard tasks completed successfully and the proportion of hard task choices (PHTC/“effort preference”). This correlation suggests that effort preference was confounded with participants' ability to complete the hard task (healthy volunteers: blue, *n* = 16; PI-ME/CFS patients: red, *n* = 15). The hard task completion rates (*x*-axis) indicate that this task was too hard for many PI-ME/CFS patients: seven of them completed it at lower rates (*M* = 0.3) than any healthy volunteer (lowest rate = 0.7).

Our own analysis of Walitt et al.'s (2024) data, accessed via mapMECFS (Mathur et al., 2021), confirmed the large difference in ability between the groups (analysis code available at https://osf.io/vqzca/). PI-ME/CFS patients were only able to complete an average of 65% of the hard tasks they chose (*SD* = 37%), compared to 96% (*SD* = 8%) for controls, two-sided Mann-Whitney *U*: *p* = 0.01, *r* = 0.45 (non-parametric analyses were used to account for extreme negative skew in completion rates). This constitutes a failure of Treadway et al.'s (2009) manipulation check for differences in ability. Moreover, as shown in Figure 1, seven of the 15 PI-ME/CFS patients had lower hard task completion rates than any control, successfully completing only 30% of the hard tasks they chose (*SD* = 23%). These results show that the hard task was simply too hard for many PI-ME/CFS patients. By contrast, both groups completed the easy task at near-ceiling rates (PI-ME/CFS: *M* = 98%, *SD* = 4%; controls: *M* = 99%, *SD* = 2%), two-sided Mann-Whitney *U*: *p* = 0.63.

The stark contrast in performance on the two tasks suggests that, for the PI-ME/CFS patients, choosing the hard task over the easy one (PHTC) may have been more a matter of ability than preference. Consistent with this possibility, we found that PHTC was positively correlated with the proportion of hard tasks completed successfully, *r*_*s*_(29) = 0.38, *p* = 0.03 (see Figure 1). These results suggest that PHTC was confounded with ability in Walitt et al.'s (2024) study: participants who had more difficulty completing the hard task chose it less often. Because difficulty with the hard task disproportionately affected the PI-ME/CFS group, the large difference in hard task completion rates could explain the comparatively small difference in PHTC between groups. Therefore, we interpret Walitt et al.'s data as showing that people with PI-ME/CFS were less able to execute the EEfRT's hard task, rather than unwilling to expend effort.

To some, it might seem implausible that those with PI-ME/CFS would be unable to complete a rapid repetitive button-pressing task despite expending effort. Yet there are at least two reasons why this finding is unsurprising. First, the hard task on the EEfRT was designed to be challenging but achievable for healthy people (Treadway et al., 2009). ME/CFS patients, however, report greater physical limitations than healthy people and those with other disabling illnesses on the Short-Form 36 (SF-36) measure of health-related quality of life, including its physical function and role-physical subscales (Komaroff et al., 1996; Nacul et al., 2011). In an analysis of Walitt et al.'s (2024) SF-36 data, we found that the PI-ME/CFS group had particularly poor physical capacity, with physical function scores (*M* = 28.7, *SD* = 21.1, *n* = 15) far below those of the control group (*M* = 97.5, *SD* = 4.1, *n* = 16; scale: 0–100), two-sided Mann-Whitney *U*: *p* < 0.001, *r* = 0.87. Second, these general physical limitations may be accompanied by more specific psychomotor deficits. Although Walitt et al. found no significant group differences on one measure of psychomotor ability (Grooved Pegboard Test; Kløve, 1963), their analyses were underpowered to detect even large differences (e.g., *d* = 0.8: 60% power). Indeed, studies using several other measures point to motor speed impairments in ME/CFS (Majer et al., 2008; Michiels et al., 1996; Schrijvers et al., 2009), echoing patients' cognitive processing speed deficits (Lange et al., 2024; Sebaiti et al., 2022). In light of their significant physical limitations and the broader body of evidence for psychomotor deficits, it is little wonder that Walitt et al.'s PI-ME/CFS patients struggled to press a button 98 times in 21 s with the non-dominant little finger.

To distinguish willingness to expend effort from ability, several investigators have calibrated the higher-effort task on the EEfRT to individual ability levels (Cooper et al., 2019; Fervaha et al., 2013; Le et al., 2023; Reddy et al., 2015). For example, in a study of people with schizophrenia, Reddy et al. (2015) used a calibration phase to determine each participant's maximum button pressing rate, and then set that participant's hard task target to 85% of their maximum. Others have suggested that the calibration phase could itself be affected by reduced willingness to expend effort (Le Bouc et al., 2023), thus artificially lowering the target for the hard task and obscuring group differences. In an attempt to mitigate this concern in a study using a hand grip task, Le Bouc et al. (2023) set hard task targets based on participants' estimated potential arm strength rather than their performed arm strength. However, this approach is insufficient because it targets only *some* of the skills required for the high-effort task. Calibration should be task-specific, targeting *all* skills required for the task. Otherwise, group differences in any number of psychomotor skills could explain the results. For this reason, there is no way to retrospectively compensate for the lack of calibration in Walitt et al.'s (2024) study. Adequate performance on other measures does not imply that participants are readily able to complete the hard task on the EEfRT.

## Conclusion

In sum, Walitt et al.'s (2024) data provide no evidence of altered effort preference in PI-ME/CFS patients, who lacked the physical ability to consistently execute the task assessing it. Conclusions about effort preference are unwarranted when group differences in ability could account for disparities in task performance. To decouple what patients are *willing* to do from what they are *able* to do, future research in ME/CFS should calibrate measures of effort-based decision-making to the ability of individual patients. The amount of effort a person wants to exert on a task is irrelevant if they are unable to exert it.

## Footnotes

^1^Neither within-task fatigue nor reward sensitivity differed between groups.

^2^Performance feedback was provided both during and after each trial. Upon completing the measure, participants won a monetary reward for two of their successful trials. Reward values and probabilities of receiving the reward changed from trial to trial, with higher-value rewards for the hard task.
